# Metabolic enzymes: key modulators of functionality in cancer stem-like cells

**DOI:** 10.18632/oncotarget.14041

**Published:** 2016-12-20

**Authors:** Bo-Wen Dong, Guang-Ming Qin, Yan Luo, Jian-Shan Mao

**Affiliations:** ^1^ Department of Gastroenterology, Second Affiliated Hospital of Zhejiang University School of Medicine, Hangzhou, China; ^2^ Department of Clinical Laboratory Medicine, Second Affiliated Hospital of Zhejiang University School of Medicine, Hangzhou, China; ^3^ School of Basic Medical Sciences, Zhejiang University, Hangzhou, China

**Keywords:** cancer stem-like cells, metabolism, enzymes, stemness, mechanism

## Abstract

Cancer Stem-like Cells (CSCs) are a subpopulation of cancer cells with self-renewal capacity and are important for the initiation, progression and recurrence of cancer diseases. The metabolic profile of CSCs is consistent with their stem-like properties. Studies have indicated that enzymes, the main regulators of cellular metabolism, dictate functionalities of CSCs in both catalysis-dependent and catalysis-independent manners. This paper reviews diverse studies of metabolic enzymes, and describes the effects of these enzymes on metabolic adaptation, gene transcription and signal transduction, in CSCs.

## INTRODUCTION

According to the hierarchical model, tumor is formed by a hierarchy of proliferative and progressively differentiated bulk of cells, which originated from a unique subpopulation of cells showing “stem-like” property, cancer stem-like cells (CSCs) [1]. CSCs share many features with normal stem cells, for example, they show self-renewal and differentiation capacity, and have extraordinary proliferation potential. A unique/novel feature of CSCs is that they have the capacity to resist chemotherapy and irradiation due to expression of multidrug resistance related transporters and DNA repair mechanisms [2, 3]. Together with their invasive and metastatic capabilities, CSCs correlate closely with tumor initiation, progression, and recurrence.

Metabolism is the basic process of material circulation and energy exchange in a life entity. At the cellular level, metabolism represents the biochemical reactions supporting the regulation of cellular bioactivities. The metabolic characteristics of cancer cells closely correlated with their malignancy. In general, cancer cells have enhanced anabolism of proteins, lipids, and nucleic acids, and they catabolize glucose in a manner of aerobic glycolysis, a.k.a. the Warburg effect [4], in order to meet the nutrient demands of malignant proliferation [5].

**Figure 1:**
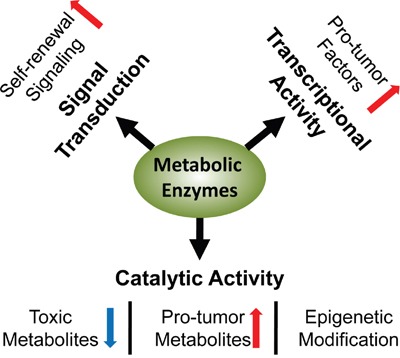
Multifaceted regulation in CSCs involving enzymes Metabolic enzymes mediate the pro-stemness metabolism by their catalytic activities, and stimulate the expression of pro-tumor factors by their transcriptional activities; they promote the self-renewal signaling by direct interaction or modulation of metabolites.

As a special subpopulation, CSCs share general features of metabolic processes with malignant cells; meanwhile, they have certain unique metabolic characteristics that are prominently adapted with their stemness. Metabolic enzymes are main regulators of cellular metabolism. In CSCs, many a metabolic enzyme shows both catalytic activities and transcriptional activities. They not only induce and maintain the metabolic characteristics, but also activate stem-like properties by transcriptional regulation or signal interaction (Figure 1). Such multiple layers of regulation carried out by metabolic enzymes indicate that these molecules can be used as potential targets for separation of CSCs, and detection and treatment of cancer diseases. This review focuses on the metabolic enzymes involved in carbohydrate, protein and lipid metabolism in CSCs, discussing their function in stemness promotion and the involved mechanisms.

### Altered aerobic glycolysis with branched metabolic pathway in CSCs

**Figure 2:**
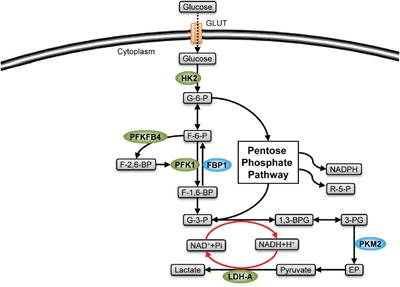
Stemness-related enzymes in glycolysis Glycolytic enzymes (green) mediate the production of glycolytic metabolites, inducing a pro-stemness metabolism by their catalytic modulation in CSCs. The enzymes shown in green are highly expressed in CSCs, while those shown in blue are expressed in low level (FBP1) or existed in low-activity form (PKM2).

Like other malignant cells, CSCs use aerobic glycolysis to satisfy their biosynthesis and energy requirement. However, some glycolytic enzymes show different expression and function in CSCs, inducing an altered aerobic glycolysis matching with stemness. Although CSCs consume more NADH and ADP to produce more glycolysis intermediates, compared with nonstem cancer cells, the two cell populations have the same ATP level [6], which means that the activated glycolysis in CSCs mainly dedicates to biosynthesis, and stimulates downstream branched metabolic pathways (Figure 2).

### Hexokinase2 (HK2)

Hexokinase is involved in the first rate-limiting reaction of glycolysis, which is the transformation of glucose to glucose-6-phosphate with ATP consumption. Among those isoenzymes of Hexokinase, Hexokinase2 (HK2) has the closest relationship with cancer. HK2 is only expressed in adipose tissue, skeletal muscle, and myocardium of normal human body at low level, but it is highly expressed in embryonic tissues and is the main isoenzyme type in cancer cells.

There are 3 main functions of HK2 in cancer cells. First, HK2 maintains the Warburg effect and its branched pathways, and the inhibition of HK2 activity will obstruct nucleotide production through the pentose phosphate pathway, disturb serine anabolism and decrease the usage of carbon derived from glutamine in the tricarboxylic acid cycle [7]. Second, HK2 combines with the Voltage Dependent Anion Channel (VDAC) on the surface of mitochondria to form a VDAC-HK2 complex, which can inhibit the formation of mitochondrial permeability transition pore, protecting the cancer cell from the release of pro-apoptosis factors induced by mitochondrial damage [8, 9]. Third, HK2 can interact with Akt/mTOR signaling pathway, regulating the growth of cancer cells [10].

HK2 plays a more important role in CSCs. The expression and enzyme activity of HK2 in CD133+ stem and CD133- nonstem cancer cells have been compared. It is shown that although HK2 is expressed less in CD133+ cells, it is almost the only type of isoenzyme, and represents 92.7% total HK enzyme activity in CD133+ CSCs [11]. Research on HK2 inhibitors revealed that they impair not only the metabolic processes, but also the stemness-related properties of CSCs. For example, 3-bromopyruvate (3-BrP), a specific HK2 inhibitor, can suppress the stemness of CSCs in two aspects. First, 3-BrP can reduce the expression of stemness-related molecules like ALDH1, Notch1, Oct-4 and Sox2, weakening CSCs’ self-renewal capacity. Second, 3-BrP can disturb the formation of VDAC-HK2 complex and block NF-κB signaling, increasing the sensitivity of CSCs to chemotherapy [12, 13]. In addition, miR-143 inhibits HK2 expression at the level of transcriptional regulation. It is demonstrated that overexpression of miR-143 can induce the differentiation of glioblastoma stem-like cells, and inhibit their tumorigenic ability under hypoxia [14]. These studies indirectly confirm the significance of HK2 in the maintenance of CSCs’ stem-like properties.

### Fructose-1,6-biphosphatase (FBP1) and Phosphofructokinase2 (PFK2)

Phosphofructokinase1 (PFK1) is the second key enzyme of glycolysis, catalyzing the transformation of fructose-6-phosphate to fructose-1,6-biphosphate. Inhibition targeting this process can strongly impair the metabolic adaptation of CSCs.

Fructose-1,6-biphosphatase (FBP1) is the key enzyme of gluconeogenesis, which catalyzes the reversed reaction and competes with branched biosynthesis pathways for substrates. Low expression of FBP1 is beneficial to CSCs. First, it maintains the superiority of glycolysis and induces an increased uptake of glucose, facilitating the production of glycolysis intermediates and the energy supply in CSCs during hypoxia. Second, low expression of FBP1 can inhibit the production of ROS induced by mitochondrial complex1, which protects CSCs from oxidative stress. The mechanism by which CSCs maintain the low expression of FBP1 is not clear. In basal-like breast cancer, it has been demonstrated that the epithelial-mesenchymal transition (EMT) factor Snail can directly bind with the promoter of the gene encoding FBP1, leading to the down regulation of FBP1 expression [15].

Different from FBP1, PFK2 catalyses the production of fructose-2,6-biphosphate, which is the most powerful allosteric activator of PFK1. PFKFB4, an isoenzyme of PFK2 family, is highly expressed in glioma CSCs. Silencing the expression of PFKFB4 by RNA interference can induce the obstruction of glycolysis, creating an increased ratio of AMP/ATP. The high AMP/ATP ratio will activate AMPK and then inhibit the mTOR-related survival signaling pathway, inducing the apoptosis of CSCs [16].

### Pyruvate kinase M2 (PKM2)

The third key enzyme of glycolysis is pyruvate kinase (PK), which catalyzes the production of pyruvate and ATP by hydrolysis of PEP. PKM2 is mainly expressed in tumor tissue and existed in a dimer form with low catalytic activity [17]. It has been proven that the PKM2 dimer form possesses special properties that maintain the stemness of CSCs.

The low activity of PKM2 is maintained mainly by phosphotyrosine signaling in CSCs. In CSCs, PKM2 will specifically bind to tyrosine-phosphorylated peptides and release its allosteric activator, fructose-1,6-biphosphate, keeping a low catalytic efficiency. What's more, CD44, a surface molecule of CSCs, can bind directly with PKM2 at its intracellular domain, synergizing the effect of tyrosine-phosphorylated peptides to maintain the low activity of PKM2, diverting glucose metabolites from energy production to biosynthesis [18, 19].

High activated tetramer form of PKM2 can also form in CSCs. The active upstream glycolysis and obstruction caused by the low activity of PKM2 will lead to the accumulation of glycolytic intermediates, including fructose-1,6-biphosphatase, the allosteric activator of PKM2, inducing the formation of tetramer to promote ATP and pyruvate production [15]. Thus, CSCs can regulate the direction of glycolysis with the variation of PKM2 activation: Low-activity form of PKM2 induces the biosynthesis of ribose and lipid by increasing the upstream glycolytic metabolites; while high-activity form accelerates the pyruvate production, facilitating energy supply and amino acid anabolism.

Apart from its metabolic regulatory function, PKM2 also acts as a transcription factor in stemness-related pathway. Induced by the activation of epidermal growth factor receptor (EGFR), PKM2 can translocate into the nucleus to activate Wnt/β-catenin signaling pathway [20]. In addition, the tetramer form of PKM2 transformed from the dimer form induced by dichloroacetate can silence Oct4 gene, leading to the differentiation of CSCs [21].

### Lactic dehydrogenase-A (LDH-A)

Lactic dehydrogenase (LDH) catalyzes the lactate production, which is the transformation of pyruvate to lactate with NAD+ production. LDH-A is mainly expressed in tumor cells. Compared with other isotypes of LDH, LDH-A can catalyze the lactate and NAD+ production more efficiently, which creates a positive feedback in the glycolytic pathway (Figure 2), and inhibits the usage of pyruvate by aerobic oxidation [22]. In non-small cell lung cancer (NSCLC), it has been proven that the lack of LDH-A will enhance the tricarboxylic acid cycle, which produces ROS and causes cell damage, decreasing both the ratio and sphere formation capacity of CSCs [23].

The regulation of pyruvate metabolism is not the only function of LDH-A in CSCs. Studies on NHI, a LDH-A specific inhibitor, show the stemness-related function of LDH in three aspects. First, LDH-A can maintain the formation of clonal spheres in 3D culture and the expression of CSC markers like CD133 and EZH2. Second, LDH-A can facilitate the expression of metalloprotease, which is important for the invasion of CSCs. Third, LDH-A dedicates to the formation of chemoresistance of CSCs, as NHI can stimulate the releasing of deoxycytidine kinase (dCK), which can enhance the anti-tumor efficiency of Gemcitabine [24].

### Bioactive lipids and related enzymes are involved in regulation of CSCs

**Figure 3:**
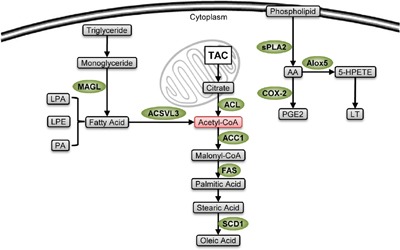
Stemness-related enzymes in lipid metabolism Lipid metabolic enzymes (green) are highly expressed in CSCs. Their lipid metabolites are used to produce membrane during cellular proliferation, coordinate biosynthesis and energetic supply through acetyl-CoA production (pink), and can maintain stem-like properties. The pro-stemness mechanisms of some of the enzymes, including MAGL, ACSVL3 and SCD1, are not fully understood.

Activated lipid metabolism is a common characteristic of tumor tissues. It can provide cell proliferation with lipid material, and produce bioactive lipids to facilitate pro-tumor signal transduction and gene expression. Many bioactive lipids and lipid metabolic enzymes can induce specific metabolic profiles to maintain CSCs’ stem-like properties (Figure 3).

### Enzymes involved in lipid synthesis

In CSCs, there is a high expression of enzymes involved in lipid synthesis including ATP citrate lyase (ACL), acetyl-CoA carboxylase1 (ACC1) and fatty acid synthase (FAS). It has been reported that expression of these enzymes is under the control of sterol regulatory element binding protein1 (SREBP1), and either chemical inhibition of these enzymes or transcriptional reduction of their gene expression can enormously impair the stem-like properties [25]. For example, inhibiting ACL by hydroxycitric acid and ACL knockdown lead the same effects including impairment of tumorsphere forming ability, reduction in the expression of stemness marker like c-Myc, Klf4, Oct4, and downregulation of the transcriptional activity of Snail [26]. A specific inhibitor of ACC1 called soraphen A shows a prominent anti-CSCs effect by inhibiting the de novo lipogenesis as it blocks the positive effect of ACC1 on survival and self-renew of CSCs from breast cancer [27]. Resveratrol, which can block the expression of fatty acid synthase, suppresses the growth of CSCs by inhibiting lipid synthesis, and induces cell death by upregulating the expression of pro-apoptosis gene DAPK2 as well as BNIP3 [28].

### Enzymes involved in PUFA metabolism

Derivatives of polyunsaturated fatty acids such as prostaglandin, thromboxane and leukotrienes are involved in many important physiological processes. Apart from regulatory roles in inflammatory responses, their influences on stem cell differentiation and proliferation facilitate malignancy development [29].

### Arachidonate 5-lipoxygenase (Alox5)

Catalyzed by lipoxygenase, arachidonic acid is converted into hydrogen peroxyeicosatetraenoic acids, which further produce bioactive lipids such as leukotrienes. In murine model of chronic myeloid leukemia induced by BCR-ABL, either transcriptional or chemical inhibition of arachidonate 5-lipoxygenase (Alox5) impairs the survival and asymmetric cell division capacity of leukemia stem cells by downregulation of β-catenin in Wnt signaling, inhibiting leukemia initiation, while the loss of Alox5 exhibits no effect on normal hematopoietic stem cells [30, 31]. Roos et al. further revealed the underlying mechanism by demonstrating that inactive form of Alox5 in association with its catalytic inhibitor CJ-13,610 impeded nuclear translocation of β-catenin by direct interaction [32]. Another inhibitor of Alox5, Nordy, has been found to decrease the expression of stemness related genes including SOX-2, Nanog and Nestin. Such change induces the formation of GFAP expressing mature glioma stem cells, and inhibits the formation of xenograft [33, 34]. These results not only show the function of Alox5 in stemness maintenance, but also indicate that anti-Alox5 therapy may be safe and effective in treatment of cancer.

### Cyclooxygenase-2 (COX-2)

Cyclooxygenase (COX) is the key enzyme in the production of prostaglandin from arachidonic acid. COX-2 is the inducible isotype involved in inflammatory responses and tumor diseases. In CSCs, high expression of COX-2 is induced by membrane type-1 matrix metalloproteinase (MT1-MMP) through MAPK/NF-κB signaling in a catalysis-independent manner [35], and is also positively correlated with macrophage migration inhibitory factor (MIF), while miRNA-451 can downregulate COX-2 expression indirectly by targeting MIF gene [36]. In breast CSCs, the expression of COX-2 also positively correlates with Her2 [37, 38]. Prostaglandin E2 catalyzed by COX-2, in conjunction with G-protein coupled receptors such as EP2, EP4 and LGR5, cause inactivation of glycogen synthase kinase 3β to decrease the inhibitory phosphorylation of β-catenin thereby enhancing β-catenin/Wnt signaling to promote the stem-like property of CSCs [39–41]. Meanwhile, COX-2 and its related metabolites can inhibit PI3K/Akt induced apoptosis by inducing the phosphorylation of Akt [42, 43]. Non-steroidal anti-inflammatory drugs (NSAIDs) like celecoxib and indomethacin can serve as COX-2 inhibitors. Apart from their inhibiting effect on COX-2 activity, NSAIDs can also inhibit Akt phosphorylation by activating PPARγ leading to amplification of PTEN signal [43, 44]. Furthermore, NSAIDs can block Wnt signaling [45]. In CSCs, it has been proven that NSAIDs can effectively inhibit tumorsphere formation and self-renewal, and impede chemoresistance [45, 46] while exhibiting no effect on the sensitivity to radiotherapy [47].

### Monoacylglycerol lipase (MAGL), Acyl-CoA synthetase VL3 (ACSVL3) and Stearoyl-coAdesaturase1 (SCD1)

There are other stemness related lipid metabolic enzymes, including Monoacylglycerol lipase (MAGL), Acyl-CoA synthetase VL3 (ACSVL3) and Stearoyl-coA desaturase1 (SCD1).

MAGL is expressed more highly in CSCs as compared with nonstem cancer cells and its upregulation is a sign of EMT [48]. MAGL catalyzes the hydrolysis of endocannabinoid 2-arachidonoylglycerol to reverse the inhibition of tumor growth and metastasis induced by endocannabinoid signaling. On the other hand, it catalyzes the production of lipid agonists of G-protein coupled receptors like lysophosphatidic acid (LA), phosphatidic acid (PA) and lysophosphatidylethanolamine (LPE), which act as pro-tumor factors.

ACSVL3 is an isoenzyme of Acyl-CoA synthetase (ACS), which catalyzes acyl-CoA production during β-oxidation of fatty acid. Activation of receptor tyrosine kinase pathways, including HGF/c-Met and EGF/EGFR pathways which promote malignant progression, leads to dramatic upregulation of ACSVL3 expression. This evidence indicates that ACSVL3 is an important metabolite highly required in CSCs [49, 50].

SCD1 is the key enzyme catalyzing synthesis of oleic acid, a monounsaturated fatty acid, from saturated fatty acid. It has been reported that SCD1 inhibitors can induce endoplasmic reticulum stress to disturb protein synthesis, and impair cytoskeleton, indicating that SCD1 is important for maintaining the stabilization of cellular structure during the survival and growth of CSCs [51, 52].

### Signal molecules as substrates of protein metabolism in CSCs

Compared with general cancer cells, CSCs have much more active protein anabolism and are more sensitive to amino acid deprivation [53]. Meanwhile, CSCs maintain cyclic utilization of amino acids and regulate their stemness signaling by regulation of proteolysis. Furthermore, amino acid metabolites promote the maintenance of stem-like properties in CSCs.

### Enzymes involved in proteolysis

**Figure 4:**
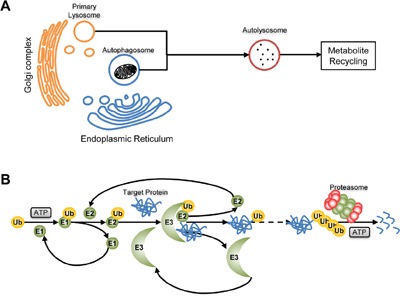
Proteolysis **A**. Cathepsin-lysosome pathway. Fusing with Cathepsin-containing primary lysosome, an autophagosome will convert to autolysosome and proteins in it will be degraded to recycle in biosynthesis. **B**. Ubiquitin-proteasome pathway. Catalyzed by E1, E2 and E3, the target protein will be modified by ubiquitin and finally degraded to peptides by proteosome.

Proteins are degraded by either the ATP-independent cathepsin-lysosome pathway or the ATP-dependent ubiquitin-proteasome pathway (Figure 4). CSCs show activated lysosome pathway to keep cyclic utilization of raw materials for protein synthesis without increasing energy demand. At the same time, they carry on proteasome pathway with low but specific activity, protecting the pro-stemness molecules while selectively degrading the signal molecules which are harmful for stem-like properties by ubiquitin modification.

### Cathepsin B

With cathepsins, lysosome can transform waste proteins to amino acid materials for protein remanufacture without ATP consumption (Figure 4A). There are natural cathepsin inhibitors in cells regulating the proteolysis activity of lysosome. One of them is called cystatin E/M that is a cathepsin B inhibitor and is expressed by CST6 gene. In CSCs, the promoter of CST6 is highly methylated and there is little cystatin E/M production. Thus, the activity of cathepsin B is maintained at high level to satisfy the active protein metabolism of CSCs [54].

Apart from metabolic regulation, Cathepsin B is also involved in other aspects in CSCs, including induction of radioresistance and maintenance of the stability of cytoskeleton.

Radiation can induce the upregulation of cathepsin B in glioma CSCs. After inhibiting such radiation-enhanced cathepsin B expression by shRNA pUC, c-Met signaling is blocked, leading to the production of DNA damage marker, γH2AX that further induces DNA damage-induced apoptosis [55]. The pUC-induced inhibition of cathepsin B also leads to disassociation of focal adhesion kinase (FAK) and cytoskeletal molecules, which further inhibits PKC/integrin signaling, impairing the adhesion and migration of CSCs [56].

### 26S Proteasome and ubiquitin chain

Proteasomes specifically recognize and degrade ubiquitinated proteins (Figure 4B). CSCs derived from glioma, breast cancer, lung cancer, hepatoma, pancreatic cancer and osteosarcoma all carry low proteasome activity. When fluorescent proteins are exogenously expressed in cancer cells, the above character induces relative accumulation of fluorescent proteins in CSCs population, which can be used for sorting of CSCs [57–62]. Musashi1, an mRNA binding protein, is the main factor maintaining the low proteasome activity of CSCs. It can downregulate the level of proteasome transcriptional factor NF-YA by directly binding to NF-YA mRNA, leading to the inhibition of proteasome production [63]. Low proteasome activity can induce a static metabolism profile in CSCs to resist stresses, and can also help CSCs to escape from immunological surveillance by reducing the production of antigen peptide [64]. In addition, proteasomes regulate the stem-like property of CSCs via degrading key factors in stemness signaling pathways. Low proteasome activity maintains the stability of transcriptional repressor REST to keep CSCs in a dedifferentiation state [65], decreases the degradation of Notch intracellular domain to sustain Notch signaling [63], and is beneficial to the stability of Nrf2 to induce the expression of anti-oxidation genes [66].

Proteasomes can also maintain stem-like properties of CSCs by specifically degrading ubiquitin modified signal molecules. Protein ubiquitination is a three-step reaction comprising ubiquitin-activating enzyme (E1), ubiquitin-conjugating enzyme (E2) and ubiquitin ligase (E3), and ubiquitin ligase is the key enzyme that catalyzes the direct covalent linkage of ubiquitin with proteins (Figure 4B). In CSCs, ubiquitin ligase supports the stemness by modifying at least three signal molecules. First, it induces the ubiquitinated degradation of receptor-interacting protein1 (RIP1) to inhibit TNFα-induced apoptosis [67]. Second, it induces the ubiquitinated degradation of cyclin E to maintain DNA stability [68]. Third, MYCN is another target of ubiquitin ligase, and its degradation can stimulate asymmetric division [69]. More importantly, many ubiquitin ligase inhibitors show anti-CSCs potentials [70–72]. Apart from ubiquitin ligase, another ubiquitination related enzyme, ubiquitin-specific protease 22, has been discovered as a CSCs marker. It can inhibit gene expression by its ubiquitin hydrolase activity to protect histone H2A and H2B from proteasome degradation [73, 74].

### Enzymes involved in amino acid catabolism

Some metabolites produced from amino acid catabolism have special effects to CSCs. And enzymes related with them promote stem-like properties of CSCs through catalyzing the production of these metabolites.

**Figure 5:**
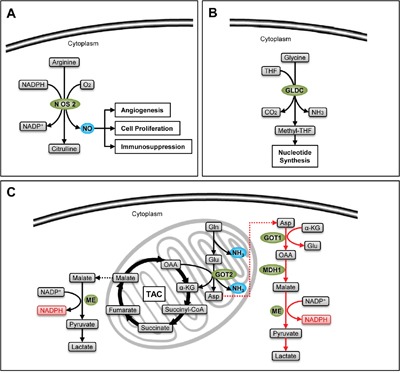
Stemness-related enzymes in amino acid catabolism **A**. NO (blue) produced by catalysis of NOS2 sustains the survival of CSCs. **B**. GLDC increases the production of one carbon units which facilitates nucleotide synthesis in CSCs. **C**. The classical glutaminolysis (black arrows) increases the TAC influx by the production of α-ketoglutarate, satisfying energetic demand, and produces NH_3_ (blue), which can neutralize acidic metabolites and induce autophagy, facilitating the survival of CSCs. Meanwhile, the non-canonical glutamine catabolism (red arrows) provides an effective compensation of NADPH (pink), protecting CSCs from oxidative stress.

Nitric oxide is produced from arginine with the catalysis of nitric oxide synthase (NOS), and is important for transduction of cell signals (Figure 5A). NOS2, also called inducible NOS (iNOS), is highly expressed in glioma CSCs (GSCs). Catalyzed by NOS2, NO inhibits the expression of the cell-cycle inhibitor cell division autoantigen-1 (CDA1) at transcriptional level to enhance the tumorigenesis capacity of GSCs. An enzymatic inhibitor of NOS2 named BYK191023 can effectively inhibit the survival of CSCs without impairing normal tissues. Because of its high selectivity, ideal pharmacokinetics, perfect bioavailability, and high permeability to pass the blood-brain barrier, BYK191023 may serve as an effective drug in glioma therapy [75].

Some amino acids can produce one carbon units essential for synthesis of purine and pyrimidine. Glycine is one of them. It produces methyl-tetrahydrofolate (methyl-THF) with the catalysis of glycine decarboxylase (GLDC) (Figure 5B). Zhang et al. detected a dramatic upregulation of GLDC in NSCLC CSCs. Interfering GLDC expression with shRNA strongly inhibit the sphere and xenograft formation capacity of CSCs, while overexpression of GLDC can give stemness to 3T3 cells. The group further demonstrated that CSCs activate glycolysis, promoting the usage of pyruvate in biosynthesis to produce amino acids, while at the same time they absorb exogenous amino acids. Such accumulation of amino acids further enhances the production of one carbon units, which satisfies the demand of nucleotides during the growth of CSCs [76].

Glutamine is the precursor of nonessential amino acids and nucleic acids. It can produce GSH and NADPH to maintain redox status, and is involved in many metabolic processes which are important for tumor growth and survival [77]. Catalyzed by glutaminase, glutamine is converted to ammonia and glutamate. In normal cells, glutamate will be further decomposed into α-ketoglutarate (α-KG) by glutamate dehydrogenase1 (GLUD1), participating in TAC, or converted into aspartate by the catalysis of glutamic oxalacetic transaminase2 (GOT2). Recently, Son et al. demonstrate another pathway of glutamine metabolism in pancreatic ductal adenocarcinoma (PDAC). They discovered that glutamate, generated from glutamine, can be converted to oxaloacetic acid by the catalysis of glutamic oxalacetic transaminase1 (GOT1) in cytoplasm, and finally becomes pyruvate with NADPH production by the catalysis of malate dehydrogenase1 (MDH1) and malic enzyme (ME). This process induces an increase of NADPH/NADP+ ratio, protecting cancer cells from oxidative stress [78] (Figure 5C). Such a non-canonical metabolic pathway of glutamine has also been found in CSCs derived from PDAC by Li et al. What's more, either inhibiting GOT1 expression or depriving glutamine can effectively suppress stem-like properties of PDAC-CSCs, and increase their sensitivity to radiotherapy by the accumulation of ROS [79]. The discovery of the non-canonical metabolic pathway of glutamine may provide new methods for PDAC therapy.

### Enzyme-associated metabolic adaptation in CSCs

**Figure 6:**
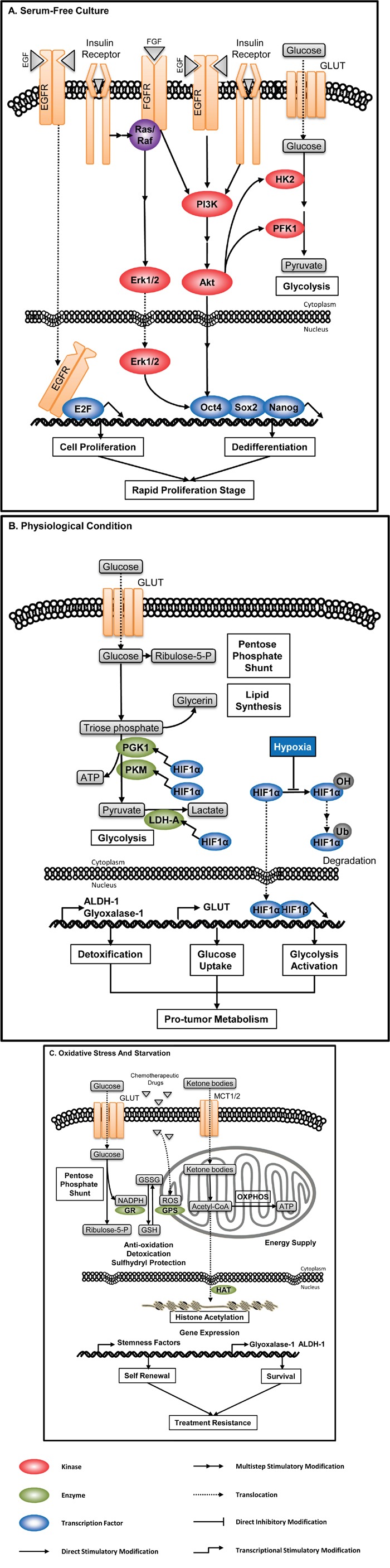
Enzyme-associated metabolic adaptation Enzyme-mediated metabolic adaptation is a dynamic process, resulting from crosstalk(s) between cellular signaling, metabolic pathways, and microenvironment. **A**. CSCs in serum-free culture. Growth factors, high glucose level and insulin in artificial culture media activate multiple signaling pathways, inducing expression of stemness-related factors and augmenting glycolytic metabolism. Thus, clonal spheres cultured from serum-free system are not ideal model to represent the metabolic profile of CSCs. **B**. CSCs under physiological hypoxia. HIF1α is a transcriptional factor activating the expression of many glycolytic enzymes including PGK1, PKM and LDH-A. High level of HIF induced by hypoxia, together with the high expression of GLUT and detoxifying enzymes, mediates the metabolic adaptation under the physiological condition. **C**. CSCs under stresses. CSCs can modulate dominant metabolic pathways to adapt different environmental stresses. When encountering chemotherapy, CSCs can increase the PPP flux to produce more NADPH, which is involved in eliminating hazardous substances such as ROS, as well as regenerating reductive GSH. Under starvation, CSCs can use ketone bodies to satisfy their energetic demand and induce epigenetic modification to survive.

The expression and activity of metabolic enzymes are highly dynamic. They are correlated with sources of tumors, growth conditions and different regulatory mechanisms [80, 81]. Enzyme-associated metabolism switch in CSCs is a dynamic process which is influenced by tumor microenvironment and stemness related signaling, reflecting the adaptation to tumor microenvironment and different stages of growth and differentiation (Figure 6).

### CSCs in serum-free culture

Activated aerobic glycolysis is the core process in the biosynthesis in CSCs. CSCs, especially of those enriched by serum-free tumorsphere culture, carry much more prominent Warburg effect than general cancer cells [82]. Serum-free culture is one of the main methods to enrich CSCs, and the tumorspheres formed are used to characterize CSCs’ properties including metabolism profiles and involved metabolic enzymes. The number and size of serum-free induced tumorspheres are main evaluation criteria of stem-like properties. The formation of clonal spheres in serum-free culture is mainly induced by exogenous growth factors, insulin and high glucose level in culture medium. These factors activate multiple pro-tumor signaling pathways. For example, growth factors activate PI3K/Akt and MAPK/Erk pathway, which can increase the catalytic activity of glycolytic kinases, stimulate cell proliferation, and maintain dedifferentiation through the activation of three key transcription factors related to pluripotency, Oct4, Sox2 and Nanog. This culture system almost totally inhibits the differentiation of CSCs, and stimulates active cell division. Thus, metabolism profiles of CSCs detected in the serum-free cultures represent the characteristic of CSCs in rapid proliferation stage (Figure 6A).

### CSCs under physiological hypoxia

Under physiological conditions, CSCs tend to accumulate in the hypoxia region of a solid tumor, and also show superiority of glycolysis. CSCs sustain rapid glycolysis judged from three aspects. First, CSCs highly express GLUT to guarantee the glucose supply [83–85]. Second, hypoxia protects the proline residue of HIF-1α from hydroxylation, thus decreasing the hydroxylation -induced ubiquitin-mediated degradation of HIF-1α, facilitating the translocation of HIF-1α into nucleus to bind with HIF-1β, which activates a series of gene expression including glycolytic enzymes like PGK1, PKM and LDH-A [24]. Third, CSCs consume metabolites rapidly with active anabolism, preventing the excessive accumulation of glycolytic products, while at the same time highly express Glyoxalase-I [86] and ALDH-1 to eliminate toxic products. The superiority of glycolysis satisfies the nutrient and ATP requirement for CSCs to maintain the growth of tumor and their own population under physiological conditions (Figure 6B).

### CSCs under stresses

The superiority of Warburg effect in CSCs is not absolute, as CSCs can regulate the balance between glycolysis, oxidative phosphorylation and pentose phosphate pathway (PPP) to resist environmental stresses (Figure 6C).

Branched pathways for biosynthesis are promoted by activated aerobic glycosis in CSCs. PPP is one of the most important processs of biosynthesis. PPP produces phosphopentose, NADPH and carbon dioxide. It can also produce fructose-6-phosphate and glyceraldehydes-3-phosphate, which participate in TAC, through a series of group-transfer reaction. PPP is significant to cancer cells. On the one hand, PPP produces phosphopentose to satisfy the demand of nucleic acid synthesis during cancer cell proliferation. On the other hand, it produces NADPH, which carries many important functions in biosynthesis: First, NADPH is the hydrogen donor in the production of lipids and amino acids. For example, NADPH provides acetyl-CoA with hydrogen atom to produce fatty acids and cholesterol, and provides hydrogen atom to α-ketoglutarate (α-KG) to produce glutamate. Second, NADPH is also involved in the hydroxylation during the production of cholesterol from squalene. Third, NADPH produced by PPP is the coenzyme of glutathione reductase. It promotes the redox of GSSG to produce GSH, protecting CSCs from ROS-induced cell damage during chemotherapy [19].

TAC in cancer cells can produce carcinogenic metabolites due to the expression of abnormal metabolic enzymes. For example, gene mutations in IDH1 and IDH2 are very common in many cancer diseases. These mutations induce the overexpression of a carcinogenic metabolite, R-2-hydroxyglutarate (R-2-HG). R-2-HG can occupy the binding site of α- ketoglutarate (α-KG) on α-KG dependent dioxygenases like histone demethylation enzymes, inducing a wide range of DNA methylations [87]. It has been proven that such modification is positively correlated with dedifferentiation and self-renewal capability of CSCs [88].

Oxidative phosphorylation, the final process of biological oxidation and the main process of ATP production, may also support stem-like properties by certain means. Bedaquiline, an antibiotic used in the therapy of multi-drug resistant pulmonary tuberculosis, have been proved to have anti-cancer activity. It can specifically bind with mitochondrial ATP-synthase in CSCs, leading to mitochondrial dysfunction and oxidative stress, while is well-tolerated in normal cells [89]. This finding indicates a correlation between chemoresistance and oxidative respiratory chain in CSCs.

It has been found that CSCs can consume extracelluar high-energy metabolites such as pyruvate, lactate and ketone bodies to produce energy through mitochondrial oxidative phosphorylation during nutrient deprivation [90]. In normal human body, ketone bodies are metabolites produced by β oxidation of fatty acids, and serve as a major source of energy in brain and muscles under starvation. Thus, the usage of ketone bodies by CSCs may be an emergency plan to survive from nutrient starvation. Ketone bodies and lactate are more than emergent fuels as they facilitate stem-like properties in many aspects. Martinez-Outschoorn et al. found that ketone bodies and lactate can promote the growth of embryonic stem cells by inducing the production of many stemness-related transcriptional factors, and transform to acetyl-CoA which promotes gene expression by participating in the acetylation of histones [91]. Lamb et al. found that CSCs from *in vitro* culture of breast cancer cell lines highly express enzymes related to β oxidation. Blocking the uptake of ketone bodies and lactate with specific MCT1/2 inhibitors effectively reduces mammosphere formation [92]. Thus, they indicated that CSCs carry both production and utilization capacity of ketone bodies. However, this phenotype requires further research because it may be barely induced by artificial culture system or just related to some specific tumor origins.

### Perspectives

Base on the knowledge about cancer stem-like cells, inhibition of CSCs is the key to treat tumor diseases and prevent tumor recurrence [93]. Compare to conventional chemotherapeutic agents, metabolic enzyme inhibitors have two significant advantages. First, enzyme inhibitors can specifically target the tumorous isoenzymes, while cause little damage to normal tissues; Second, many enzyme inhibitors have been shown to cause direct damage to CSCs or impair their chemoresistance. A case study in a cancer patient with fibrolamellar hepatocellular carcinoma shows the clinical value of 3-bromopyruvate (3-BrP), a HK2 inhibitor targeting the glucose metabolism of cancer cells. TACE treatment with specially formulated 3-BrP efficiently eliminate cancer cells without apparent cyto-toxicity, lengthening the survival time of the patient with an improved life quality [94]. Studies on the CSCs specific metabolic enzymes would help find new targets for CSCs sorting and anti-tumor treatment. Meanwhile, detecting the enzymatic activities and tracing the metabolites can reflect the activity of CSCs in tumor tissues, facilitating the prognostic evaluation of tumor diseases.

## Abbreviations

3-BrP: 3-bromopyruvate
α-KG: α-ketoglutarate
ACL: ATP citrate lyase
ACS: Acyl-CoA synthetase
Alox5: arachidonate 5-lipoxygenase
CDA1: cell division autoantigen-1
COX: cyclooxygenase
CSCs: cancer stem-like cells
EMT: epithelial-mesenchymal transition
FAK: focal adhesion kinase
FBP1: fructose-1,6-biphosphatase
GLDC: glycine decarboxylase
GLUD1: glutamate dehydrogenase1
GOT1: glutamic oxalacetic transaminase1
GOT2: glutamic oxalacetic transaminase2
HK2: hexokinase2
IDH: isocitrate dehydrogenase
iNOS: inducible nitric oxide synthase
LA: lysophosphatidic acid
LDH: lactic dehydrogenase
LPE: lysophosphatidyl ethanolamine
MAGL: monoacylglycerol lipase
MCT: monocarboxylate transporter
MDH1: malate dehydrogenase1
ME: malic enzyme
MIF: macrophage migration inhibitory factor
MT1-MMP: membrane type-1 matrix metalloproteinase
NOS: nitric oxide synthase
NSAIDs: non-steroidal anti-inflammatory drugs
NSCLC: non-small cell lung cancer
PA: phosphatidic acid
PDAC: pancreatic ductal adenocarcinoma
PFK1: phosphofructokinase1
PK: pyruvate kinase
PPP: pentose phosphate pathway
R-2-HG: R-2-hydroxyglutarate
SCD1: stearoyl-coA desaturase1
SREBP1: sterol regulatory element binding protein1
TAC: tricarboxylic acid cycle
THF: tetrahydrofolate
VDAC: voltage dependent anion channel.
